# Assessing the gastrointestinal and psychological impacts of nicotine pouch use among adults in Saudi Arabia: A cross-sectional study

**DOI:** 10.18332/tpc/207753

**Published:** 2025-08-18

**Authors:** Amal M. Al-Nafisi, Ziyad B. Alsulami, Musab B. Alsulami, Ali K. Alhazmi, Bassam A. AlKhrashi, Meshal A. Alzakari, Alwaleed S. Alshutwi, Khaled Y. Bokhari, Faisal Alzkari, Osama T. Al-Ahmari

**Affiliations:** 1 Department of Public Health, College of Medicine, Imam Mohammad Ibn Saud Islamic University (IMSIU), Riyadh, Saudi Arabia

**Keywords:** nicotine pouch use, gastrointestinal, psychological impact, KSA

## Abstract

**INTRODUCTION:**

Nicotine pouches have recently gained popularity among adults in Saudi Arabia, but limited information is available about the common symptoms’ users may experience. This study looks into how frequently users report gastrointestinal and psychological symptoms and explores possible links with different personal and behavioral factors.

**METHODS:**

We conducted an online cross-sectional survey among adult Saudis who used nicotine pouches in the recent half year. The survey collected demographic data, patterns of usage, and gastrointestinal symptoms self-assessed. Psychiatric status was assessed using the Arabic DASS-21 version. Data were analyzed using SPSS version 29 with the help of descriptive and logistic regression analysis.

**RESULTS:**

A total of 1214 individuals participated. Most (80.8%) reported at least one gastrointestinal symptom – mainly bloating (66.7%), nausea (47.9%), and heartburn (46.7%). Around 39.3% of participants reported psychological symptoms, with mild symptoms being the most common (19.9%). The analysis showed that people with lower education and income levels were more likely to report psychological symptoms (p=0.004 and p<0.001). A slight trend was also noted among current smokers, though not statistically significant (p=0.076).

**CONCLUSIONS:**

The study found that many users of nicotine pouches report gastrointestinal and psychological symptoms. The symptoms seem more common among individuals with lower socioeconomic status. Although the findings do not imply direct effects, they indicate that there should be greater awareness and more research, especially long-term research, to establish how nicotine pouch use can be attributed to health problems.

## INTRODUCTION

Nicotine and its harmful effects have been widely known in matters of both psychological and gastrointestinal (GI) health. It is associated with a number of gastrointestinal (GI) problems like Crohn disease, functional dyspepsia, gastroesophageal reflux disease, and chronic pancreatitis^1^. Because of its addictive features, nicotine has also been connected to depression, anxiety, and stress^2^. Additionally, tobacco-free nicotine products, such as nicotine pouches, can help individuals quit or decrease their tobacco consumption, potentially decreasing the symptoms of its unfavorable effect. Therefore, they are marketed as a lower risk alternative or method of quitting smoking; however, their long-term health effects are not entirely understood in Saudi Arabia. Nicotine pouches are small pouches consisting of nicotine but no tobacco leaf and, in each sachet, hold flavored crystalline nicotine, different salts, and cellulose, with nicotine strengths typically ranging from 3 to 10 mg. Unlike traditional tobacco products, they do not require saliva for use. They are placed enclosed by their lips and gums for 30 minutes, as they are absorbed through the oral mucosa to infiltrate the blood system with nicotine^3^. Because of the small size and fast delivery of nicotine, there is a growing demand for using nicotine pouches, increasing the addiction to nicotine. This trend raises worries about the possibility of increased oral nicotine pouch impact on gastrointestinal (GI) disorders and psychiatric change; therefore, oral nicotine pouches are still a relatively new product in Saudi Arabia, and their effects on mental and digestive health are not well understood yet. Unlike cigarettes, which have been investigated so extensively over the years, these pouches have received little attention by scientists. But a 2023 report by Dowd et al.^4^ provides some preliminary findings. Of 118 adult users, almost half had oral lesions. Others had stomach upset (39%), sore mouth (37%), sore throat (21%), and nausea (9%). These findings highlight the importance of research into the potential health risks of such products, particularly their impact on digestion and overall physical health^4^. In addition to its physical effects, the active ingredient in nicotine pouches has been increasingly associated with mental health concerns. For instance, a 2022 study by Sushanthi et al.^2^ examined 416 male construction workers in Chennai, India, and found a significant link between nicotine dependence and symptoms of anxiety and depression. The study employed standardized tools such as the GAD-7, PHQ-9, and the Fagerström test for nicotine dependence to assess these associations^2^. Oral nicotine pouches, however, are not the same as traditional tobacco products. Different health outcomes may result from their special delivery method and additional ingredients. How they impact the gastrointestinal system, including aspects like gut motility, bloating, or long-term digestive issues, is still largely unknown. Similarly, more focused studies are required to determine their effects on mental health. The gap is particularly significant in Saudi Arabia, where social norms and cultural practices may influence how these products are used and how people respond to them.

### Study design

The was a cross-sectional study with the objective of exploring the gastrointestinal and psychological effects of nicotine pouch consumption in Saudi Arabian adults.

### Setting

The study was conducted online using an online self-administered questionnaire developed through Google Forms. Data were collected in the period 2–7 January 2025, and the survey was conducted via Telegram groups Saudi Arabian consumers of nicotine pouches utilized.

### Participants

Adults aged ≥18 years who were residents in Saudi Arabia and had used nicotine pouches in the past six months were recruited. We excluded minors aged <18 years, non-residents, first-time or occasional users, and any individual with a pre-existing psychological or gastrointestinal condition, in an effort to keep the sample targeted and reduce bias.

### Data sources and measurement

The questionnaire had three parts. The first part asked about general demographics like age, gender, education level, income, and where they lived. The second part asked about nicotine pouch use – how long they had been using them, how often, and what strength they used. The third part asked about health effects. For gastrointestinal symptoms like nausea, bloating, and heartburn, we used simple questions based on tools from prior research. For psychological symptoms, we used the Arabic version of the DASS-21 scale, a validated questionnaire to assess depression, anxiety, and stress levels. All questions were mandatory to avoid missing data.

### Bias

Steps to reduce bias were done by anonymizing responses, using a validated tool, and excluding individuals with known comorbidities that might complicate the results.

### Study size

A minimum sample size of approximately 380 participants was calculated based on standard cross-sectional study parameters. However, we collected a larger sample (n=1214) to enhance the reliability of the findings and reduce potential bias.

### Statistical analysis

Data analysis used SPSS Statistics version 29. Descriptive statistics were used to present the data, and bivariate and multivariate logistic regression analysis was used to find associations of nicotine pouch use with gastrointestinal or psychological symptoms. However, multivariate analysis was only performed for psychological symptoms. Adjusted odds ratios (AORs) and 95% confidence intervals (CIs) were reported, and a p<0.05 was considered statistically significant.

### Ethical approval

Ethical approval for the current study was obtained from Imam Mohammad Ibn Saud Islamic University Institutional Review Board with project number 724/2024 and registration number HAPO-01-R-061. Informed electronic consent was provided to all participants prior to their participation in the survey.

## RESULTS

A total of 1214 participants were included in the study. The majority were aged 24–40 years (920 participants; 75.8%), and the overwhelming majority were male (1207; 99.4%). More than half of the participants (670; 55.2%) held a Bachelor’s degree, and a similar proportion (638; 52.6%) reported a monthly income between 5000 and 15000 Saudi Riyals. Approximately one-third (366; 30.1%) were residents of the Mecca province (Table 1).

**Table 1:** Sociodemographic characteristics of the participants (N=1214)

*Characteristics*	*Category*	*n (%)*
**Age** (years)	18–24	185 (15.2)
	24–40	920 (75.8)
	40–65	109 (9.0)
**Sex**	Female	7 (0.6)
	Male	1207 (99.4)
**Education level**	Bachelor’s	670 (55.2)
	Diploma	267 (22.0)
	High school	202 (16.6)
	Postgraduate studies	75 (6.2)
**Monthly income** (SAR)	<5000	324 (26.7)
	5000–15000	638 (52.6)
	15001–25000	198 (16.3)
	>25000	54 (4.4)
**Province**	Al Madinah Al Munawwarah	86 (7.1)
	Al-Baha	22 (1.8)
	Al-Jawf	3 (0.2)
	Al-Qassim	30 (2.5)
	Aseer	121 (10.0)
	Eastern	147 (12.1)
	Hail	11 (0.9)
	Jizan	39 (3.2)
	Macca	366 (30.1)
	Najran	28 (2.3)
	Northern border	10 (0.8)
	Riyadh	317 (26.1)
	Tabuk	34 (2.8)

SAR: 1000 Saudi Riyals about US$270.

**Figure 1:**
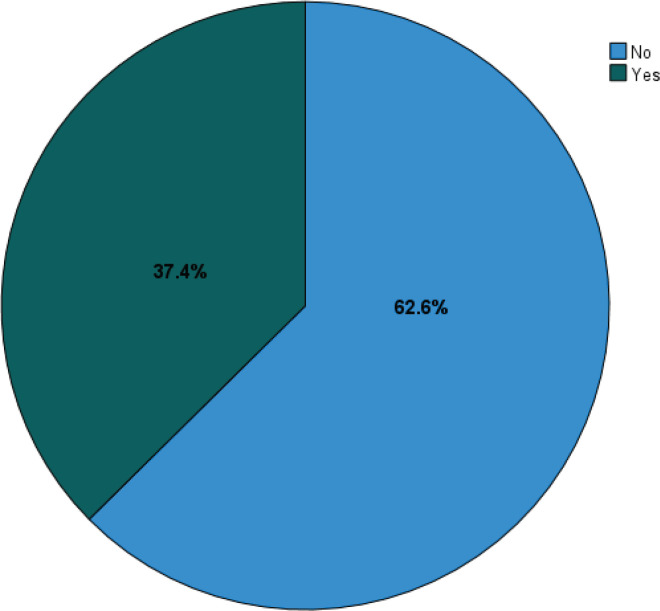
Smoking prevalence among the participants

Figure 1 illustrates the smoking prevalence among participants. Of the 1214 participants, 454 (37.4%) were smokers, while the majority, 760 (62.6%), were non-smokers.

Table 2 presents data on participants’ use of nicotine pouches. Of the 454 smokers, 244 participants (20.1%) were constant smokers, while 210 (17.3%) smoked intermittently. More than half of the participants (634; 52.2%) were ex-smokers. Among nicotine pouch users, the majority (367; 80.8%) reported using them at any time. Nearly half (191; 42.1%) had used nicotine pouches for a period of one month to less than six months. An overwhelming majority (429; 94.5%) used the 10 mg strength pouch, and most users (330; 72.7%) reported using nicotine pouches more than once a week.

**Table 2:** Participants’ nicotine pouch use

*Nicotine use status*	*Category*	*n (%)*
**Smoking**	Yes, I smoke constantly	244 (20.1)
	Yes, I smoke now and then	210 (17.3)
	No, ex-smoker	634 (52.2)
	I don’t smoke	126 (10.4)
**Time of use of nicotine pouch**	After meals	36 (7.9)
	Before bed	5 (1.1)
	There is no certain time, at any time	367 (80.8)
	When I wake up	12 (2.6)
	While working/studying	34 (7.5)
**Duration of use of nicotine pouch**	Less than a month	38 (8.4)
	From one month to less than six months	191 (42.1)
	From six months to less than a year	132 (29.1)
	More than a year	93 (20.5)
**Nicotine strength used from nicotine pouch**	10 mg (strong)	429 (94.5)
	3 mg (mild)	51 (0.2)
	6 mg (medium)	13 (2.9)
	Don’t use a specific strength	11 (2.4)
**Frequency of use of nicotine pouch**	Daily	102 (22.5)
	More than once a day	330 (72.7)
	Sometimes (between now and then)	19 (4.2)
	Weekly	3 (0.7)

The results showed a statistically significant association between participants’ age and their frequency of nicotine pouch use (p=0.020). A majority of participants aged 18–24 years reported frequent use (150; 81.1%). In contrast, most participants aged 24–40 years reported regular use (246; 26.7%) or occasional use (31; 3.4%). However, no statistically significant associations were found between nicotine pouch use and participants’ sex, education level, monthly income, or province of residence (p=0.706, p=0.957, p=0.226, and p=0.215, respectively) (Table 3).

**Table 3:** The association between sociodemographic factors and participants’ nicotine pouch use

*Variables*	*Category*	*Frequently*	*Regularly*	*Occasionally*	*p*
**Age** (years)	18–24	150 (81.1)	31 (16.8)	4 (2.2)	0.020*
	24–40	643 (69.9)	246 (26.7)	31 (3.4)	
	40–65	85 (78.0)	22 (20.2)	2 (1.8)	
**Sex**	Female	6 (85.7)	1 (14.3)	0 (0.0)	0.706
	Male	872 (72.2)	298 (24.7)	37 (3.1)	
**Education level**	Bachelor’s	491 (73.3)	158 (23.6)	21 (3.1)	0.957
	Diploma	187 (70.0)	72 (27.0)	8 (3.0)	
	High school	144 (71.3)	52 (25.7)	6 (3.0)	
	Postgraduate study	56 (74.7)	17 (22.7)	2 (2.7)	
**Monthly income** (SAR)	<5000	233 (71.9)	78 (24.1)	13 (4.0)	0.226
	5000–15000	460 (72.1)	162 (25.4)	16 (2.5)	
	15001–25000	151 (76.3)	43 (21.7)	4 (2.0)	
	>25000	34 (63.0)	16 (29.6)	4 (7.4)	
**Province**	Al Madinah Al Munawwarah	68 (79.1)	17 (19.8)	1 (1.2)	0.215
	Al-Baha	17 (77.3)	3 (13.6)	2 (9.1)	
	Al-Jawf	3 (100.0)	0 (0.0)	0 (0.0)	
	Al-Qassim	21 (70.0)	6 (20.0)	3 (10.0)	
	Aseer	97 (80.2)	23 (19.0)	1 (0.8)	
	Eastern	110 (74.8)	36 (24.5)	1 (0.7)	
	Hail	7 (63.6)	4 (36.4)	0 (0.0)	
	Jizan	27 (69.2)	11 (28.2)	1 (2.6)	
	Macca	258 (70.5)	94 (25.7)	14 (3.8)	
	Najran	22 (78.6)	6 (21.4)	0 (0.0)	
	Northern border	5 (50.0)	5 (50.0)	0 (0.0)	
	Riyadh	219 (69.1)	85 (26.8)	13 (4.1)	
	Tabuk	24 (70.6)	9 (26.5)	1 (2.9)	

* Significant at p<0.05 level.

**Figure 2:**
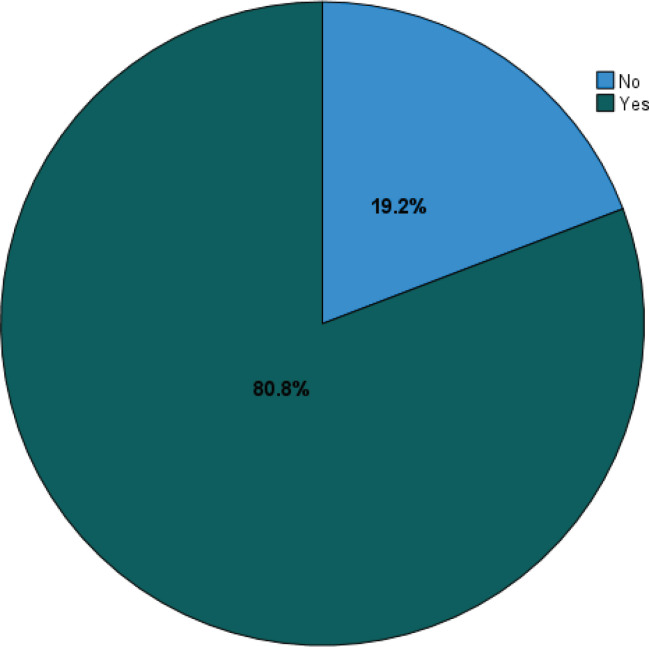
Prevalence of gastrointestinal symptoms

Figure 2 illustrates the prevalence of gastrointestinal symptoms reported by participants following nicotine pouch use. Of the 1214 participants, 981 (80.8%) reported gastrointestinal symptoms, while only 233 participants (19.2%) reported no symptoms of gastrointestinal symptoms.

**Figure 3:**
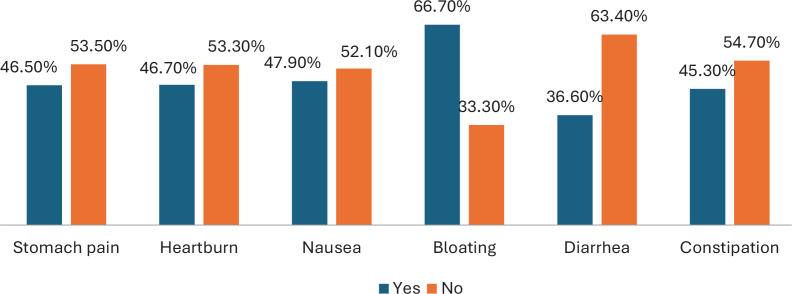
Gastrointestinal symptoms

Figure 3 shows that the most commonly reported symptom was bloating (66.7%), followed by nausea (47.9%), heartburn (46.7%), stomach pain (46.5%), and constipation (45.3%), with diarrhea being the least reported (36.6%).

The results showed statistically significant associations between participants’ education level and monthly income with the prevalence of gastrointestinal symptoms (p<0.001 and p=0.024, respectively). A higher proportion of participants with a diploma education level (83.5%) and those earning <5000 SAR per month (83.6%) reported gastrointestinal symptoms compared to their counterparts. However, no statistically significant associations were observed between other participant characteristics and the prevalence of gastrointestinal symptoms (Table 4).

**Table 4:** The association between participants’ attributes and the prevalence of gastrointestinal symptoms

*Variables*	*Category*	*No n (%)*	*Yes n (%)*	*p*
**Age** (years)	18–24	28 (15.1)	157 (84.9)	0.171
	24–40	179 (19.5)	741 (80.5)	
	40–65	26 (23.9)	83 (76.1)	
**Sex**	Female	0 (0.0)	7 (100.0)	0.196
	Male	233 (19.3)	974 (80.7)	
**Education level**	Bachelor’s	115 (17.2)	555 (82.8)	<0.001*
	Diploma	44 (16.5)	223 (83.5)	
	High school	48 (23.8)	154 (76.2)	
	Postgraduate study	26 (34.7)	49 (65.3)	
**Monthly income** (SAR)	<5000	53 (16.4)	271 (83.6)	0.024*
	5000–15000	128 (20.1)	510 (79.9)	
	15001–25000	34 (17.2)	164 (82.8)	
	>25000	18 (33.3)	36 (66.7)	
**Current smoking**	Yes	83 (18.3)	371 (81.7)	0.533
	No	150 (19.7%)	610 (80.3)	
**Smoking patterns**	Smoking constantly	45 (18.4)	199 (81.6)	0.825
	Smoke now and then	38 (18.1)	172 (81.9)	
	No, ex-smoker	128 (20.2)	506 (79.8)	
	I don’t smoke	22 (17.5)	104 (82.5)	
**Frequency of use**	Frequently	172 (19.6)	706 (80.4)	0.844
	Regularly	54 (18.1)	245 (81.9)	
	Occasionally	7 (18.9)	30 (81.1)	
**Time of use of nicotine pouch**	After meals	25 (22.1)	88 (77.9)	0.720
	Before bed	1 (10.0)	9 (90.0)	
	There is no certain time, at any time	192 (18.8)	828 (81.2)	
	When I wake up	4 (16.0)	21 (84.0)	
	While working/studying	11 (23.9)	35 (76.1)	
**Duration of use of nicotine pouch**	Less than a month	19 (31.1)	42 (68.9)	0.103
	From one month to less than six months	90 (19.2)	379 (80.8)	
	From six months to less than a year	69 (17.8)	319 (82.2)	
	More than a year	55 (18.6)	241 (81.4)	
**Nicotine strength used**	10 mg (strong)	214 (18.9)	917 (81.1)	0.218
	3 mg (mild)	2 (40.0)	3 (60.0)	
	6 mg (medium)	14 (26.9)	38 (73.1)	
	Don’t use a specific strength	3 (11.5)	23 (88.5)	

* Significant at p<0.05 level.

**Figure 4:**
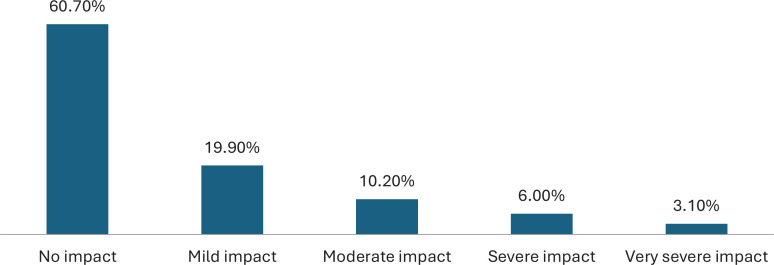
Levels of psychological impact of nicotine pouch use

Figure 4 illustrates the levels of psychological impact reported by participants following nicotine pouch use. A total of 477 participants (39.3%) reported some psychological impact, while the majority, 737 participants (60.7%), reported no impact. Among those who reported psychological impact, mild impact was the most common (242; 19.9%), followed by moderate impact (124; 10.2%), severe impact (73; 6.0%), and very severe impact (38; 3.1%).

The results showed statistically significant associations between participants’ level of education, monthly income, smoking patterns, and the psychological impact of nicotine pouch use (p=0.002, p<0.001, and p=0.015, respectively). A higher proportion of participants with a diploma education level (44.6%), those with a monthly income <5000 SAR (48.1%), and those who smoked nicotine pouches constantly (43.9%) reported experiencing psychological impacts. However, no statistically significant associations were found between other participant characteristics and the psychological impact of nicotine pouch use (Supplementary file Table 1).

The results of the multiple logistic regression analysis (Table 5) identified education level (AOR=0.83; 95% CI: 0.727–0.940; p=0.004) and monthly income (AOR=0.72; 95% CI: 0.608–0.853; p<0.001) as significant factors associated with psychological impact following nicotine pouch use. Although current smoking showed a positive association (AOR=1.25; 95% CI: 0.977–1.590), this finding was not statistically significant (p=0.076) (Table 5).

**Table 5:** Factors associated with psychological impact of nicotine pouch use

*Variables*	*AOR*	*95% CI*	*p*
Age (years)	1.02	0.783–1.317	0.909
Sex	1.13	0.251–5.098	0.873
Education level	0.83	0.727–0.940	0.004*
Monthly income	0.72	0.608–0.853	<0.001*
Current smoking	1.25	0.977–1.590	0.076
Frequency of use of nicotine pouch	0.94	0.750–1.175	0.579
Time of use of nicotine pouch	1.02	0.867–1.193	0.841
Duration of use of nicotine pouch	1.12	0.977–1.278	0.104
Nicotine strength used	1.03	0.810–1.306	0.818

AOR: adjusted odds ratio.

* Significant at p<0.05 level.

## DISCUSSION

Nicotine pouches are slowly gaining acceptance as a socially convenient alternative nicotine product, largely due to their perceived safety and social convenience. They are perceived by most people as safer than traditional cigarettes since they do not involve tar, smoke, or burning^4,5^. Even though their use has been growing in popularity, the potential health effects of nicotine pouches, such as gastrointestinal and psychological disturbances, remain an issue, particularly in some countries such as Saudi Arabia, where there has been limited research. The present study aims to fill this knowledge gap by exploring the prevalence of gastrointestinal symptoms and psychological effects of nicotine pouch use among Saudi Arabian adults. The study proved that 981 (80.8%) of the participants experienced gastrointestinal symptoms, much higher than the 64.6% reported by Farooqi et al.^6^ among electronic cigarette users in Riyadh, Saudi Arabia. The rising trend illustrates the potentiality of harm related to nicotine commodities and the need to direct awareness campaigns towards informing the public about their danger to health as well as providing preventive measures. The most commonly reported gastrointestinal symptoms following the use of nicotine pouches were bloating (66.7%), followed by nausea (47.9%), heartburn (46.7%), and abdominal pain (46.5%). The findings agree with those of Debnath et al.^7^ who reported nausea and gastric burning as common gastrointestinal symptoms among nicotine users, highlighting the gastrointestinal side effects associated with nicotine. With regard to frequency of nicotine pouch use, the majority of participants (878; 72.3%) reported using them frequently. Young adults aged 18–24 years (150; 81.1%) comprised the largest proportion of these frequent users. This finding is consistent with a Saudi study by Aldhahir et al.^8^ in which nicotine pouch use was also highest among the young age groups, particularly those aged 18–34 years. The current study established that 477 (39.3%) participants had some level of psychological effect, and the majority (737; 60.7%) had none. The most common to have experienced was mild impact (242; 19.9%), then moderate impact (124; 10.2%). Another Saudi study by Albarrak et al.^9^ reported that 42.4% of e-cigarette smokers had mild anxiety and 36.7% mild depression. The lower psychological effects in our study indicate a greater mental health burden and imply the likelihood of progression to more serious symptoms if not treated at an initial stage.

Although previous studies by Rungraungrayabkul et al.^10^ and Alizadehgharib et al.^11^ reported no serious adverse health effects of nicotine pouch use, our findings show potential side effects, namely psychological effects. The fact that such a large proportion of the sample reported mild to moderate psychological symptoms points to the need for more studies on the safety and long-term effects of nicotine pouch use. The study found education level (AOR=0.83; 95% CI: 0.727–0.940; p=0.004) and monthly income (AOR=0.72; 95% CI: 0.608–0.853; p<0.001) to be the determinants of psychological effect following the use of nicotine pouches. The findings explain that higher levels of education and income are associated with a reduced likelihood of experiencing psychological effects. This identifies the role education and socioeconomic status may play, perhaps through increased health awareness in averting psychological issues related to nicotine pouch consumption.

### Limitations

The limitations of the study should be carefully considered in the interpretation of the findings. While the cross-sectional design facilitated identification of associations between variables, it does not permit conclusions on causality. Additionally, the data collection process through online Google Forms may have been prone to recall bias and social desirability bias in the case of the participants’ responses on smoking status and frequency. These elements could have impacted the accuracy and reliability of the findings.

## CONCLUSIONS

The study revealed a very high incidence of gastrointestinal symptoms following the use of nicotine pouches among Saudi Arabian adults, with nausea and bloating being the most common symptoms. The users also had mild to moderate psychological effects, and education level and monthly income were significant variables that were associated with the psychological effects. These findings highlight the significance of increased awareness of health in order to promote increased knowledge of the potential harm of nicotine pouch use and specific interventions to deal with the increasing public health concern because of its growing consumption in Saudi Arabia.

## Supplementary Material



## Data Availability

The data supporting this research are available from the authors on reasonable request.
